# BiP Heterozigosity Aggravates Pathological Deterioration in Experimental Amyotrophic Lateral Sclerosis

**DOI:** 10.3390/ijms222212533

**Published:** 2021-11-20

**Authors:** Marta Gómez-Almería, Sonia Burgaz, Carlos Costas-Insua, Carmen Rodríguez-Cueto, Irene Santos-García, Ignacio Rodríguez-Crespo, Concepción García, Manuel Guzmán, Eva de Lago, Javier Fernández-Ruiz

**Affiliations:** 1 Instituto Universitario de Investigación en Neuroquímica, Departamento de Bioquímica y Biología Molecular, Facultad de Medicina, Universidad Complutense, 28040 Madrid, Spain; margom27@ucm.es (M.G.-A.); soniabur@ucm.es (S.B.); carc@med.ucm.es (C.R.-C.); isantosg@ucm.es (I.S.-G.); conchig@med.ucm.es (C.G.); elagofem@ucm.es (E.d.L.); 2 Centro de Investigación Biomédica en Red de Enfermedades Neurodegenerativas (CIBERNED), 28040 Madrid, Spain; cacostas@ucm.es (C.C.-I.); jirodrig@quim.ucm.es (I.R.-C.); mguzman@quim.ucm.es (M.G.); 3 Instituto Ramón y Cajal de Investigación Sanitaria (IRYCIS), 28040 Madrid, Spain; 4 Instituto Universitario de Investigación en Neuroquímica, Departamento de Bioquímica y Biología Molecular, Facultad de Biología, Universidad Complutense, 28040 Madrid, Spain

**Keywords:** cannabinoids, CB_1_ receptors, BiP interactor protein, BiP^+/−^ mice, mSOD1 mice, amyotrophic lateral sclerosis, spinal cord, Parkinson’s disease

## Abstract

In the present study, we investigated the involvement of the chaperone protein BiP (also known as GRP78 or Hspa5), a master regulator of intracellular proteostasis, in two mouse models of neurodegenerative diseases: amyotrophic lateral sclerosis (ALS) and Parkinson’s disease (PD). To this end, we used mice bearing partial genetic deletion of the BiP gene (BiP^+/−^ mice), which, for the ALS model, were crossed with mutant SOD1 (mSOD1) transgenic mice to generate mSOD1/BiP^+/−^ double mutant mice. Our data revealed a more intense neurological decline in the double mutants, reflected in a greater deterioration of the neurological score and rotarod performance, with also a reduced animal survival, compared to mSOD1 transgenic mice. Such worsening was associated with higher microglial (labelled with Iba-1 immunostaining) and, to a lesser extent, astroglial (labelled with GFAP immunostaining) immunoreactivities found in the double mutants, but not with a higher loss of spinal motor neurons (labelled with Nissl staining) in the spinal cord. The morphological analysis of Iba-1 and GFAP-positive cells revealed a higher presence of activated cells, characterized by elevated cell body size and shorter processes, in double mutants compared to mSOD1 mice with normal BiP expression. In the case of the PD model, BiP^+/−^ mice were unilaterally lesioned with the parkinsonian neurotoxin 6-hydroxydopamine (6-OHDA). In this case, however, we did not detect a greater susceptibility to damage in mutant mice, as the motor defects caused by 6-OHDA in the pole test and the cylinder rearing test, as well as the losses in tyrosine hydroxylase-containing neurons and the elevated glial reactivity (labelled with CD68 and GFAP immunostaining) detected in the substantia nigra were of similar magnitude in BiP^+/−^ mice compared with wildtype animals. Therefore, our findings support the view that a dysregulation of the protein BiP may contribute to ALS pathogenesis. As BiP has been recently related to cannabinoid type-1 (CB_1_) receptor function, our work also opens the door to future studies on a possible link between BiP and the neuroprotective effects of cannabinoids that have been widely reported in this neuropathological context. In support of this possibility, preliminary data indicate that CB_1_ receptor levels are significantly reduced in mSOD1 mice having partial deletion of BiP gene.

## 1. Introduction

Phytocannabinoids, the active constituents of Cannabis plant, as well as endocannabinoids and synthetic cannabinoids, have been proposed as promising neuroprotective agents, a property derived from their pleiotropism and ability to activate numerous cytoprotective targets within the endocannabinoid system, but also outside this signaling system (reviewed in [1]). Such neuroprotective potential has been preclinically investigated in accidental brain damage (e.g., stroke, brain trauma, spinal injury) and, in particular, in chronic progressive disorders (e.g., Alzheimer’s disease, amyotrophic lateral sclerosis (ALS), Parkinson’s disease (PD), Huntington’s disease, and others) (reviewed in [2,3]).

An important part of these neuroprotective properties described for cannabinoids have been related to the activation of the type-1 cannabinoid (CB_1_) receptor (reviewed in [2,3]). This receptor is predominantly located in neurons in the CNS, which facilitates its role in the control of excitotoxic damage in glutamatergic neurons [4], as well as a possible contribution in the autophagy-mediated elimination of protein aggregates [5]. Data supporting CB_1_ receptor-mediated neuroprotective effects have been collected in experimental models of Alzheimer’s disease [6,7,8], Huntington’s disease [4,9,10,11,12], multiple sclerosis [13,14], PD [15,16,17], and ALS [18,19,20]. In this last disorder, CB_1_ receptor activation was found to protect murine spinal cord neurons in vitro against kainate-induced neurotoxicity [18]. Interestingly, this receptor experiences an important reduction in spinal motor neurons in the classic murine model of ALS, based on the G93A mutation of the *SOD1* gene, thus predisposing these neurons to excitotoxicity [19], a result also found in a further study measuring CB_1_ receptor sensitivity with neurophysiological recordings in the same experimental model [20]. In the case of PD, studies conducted in CB_1_ receptor knockout mice lesioned with the parkinsonian neurotoxin 6-hydroxydopamine (6-OHDA), proved a worsening in the progression of motor defects, as well as in the development of L-DOPA-induced dyskinesia compared to wildtype mice [16]. Additional studies revealed benefits with CB_1_ receptor activation against inflammatory events in MPTP-lesioned mice [15].

Despite the relevance of the CB_1_ receptor in the neuroprotective properties of cannabinoids, the identification of the specific cellular, subcellular, and molecular mechanisms underlying this therapeutic potential is still hampered, at least in part, by the lack of knowledge on the neuron subpopulation selectivity of CB_1_ receptor action. CB_1_ receptor action may be modulated in different manners, being conceivably, among different possibilities, its association to different cytoplasmic proteins through its intracellular domains, in particular its large cytoplasmic C-terminal domain. This includes, for example, the cannabinoid receptor-interacting protein 1a (CRIP1a), β-arrestins, or GPCR-associated sorting protein (GASP) [21]. Recently, we have purified the CB_1_ receptor C-terminal domain and have conducted yeast two-hybrid experiments aimed at finding new receptor interactors [22]. This approach rendered a potential CB_1_ receptor-interacting protein that may be particularly attractive owing to its involvement in neurodegenerative processes: the molecular chaperone binding immunoglobulin protein (BiP), also known as 78-kDa glucose-regulated protein (GRP78) or 70-kDa heat shock protein 5 (Hspa5). This protein plays a dual role in endoplasmic reticulum stress by controlling protein folding to prevent aggregation and also by regulating the unfolded protein response (UPR) [23,24,25]. Its dysregulation has been related to different physiopathological conditions, including physiological and pathological brain aging, for which a reduced BiP function has been proposed as a predisposing factor in different chronic neurodegenerative pathologies [23].

In the present study, we investigated the involvement of a dysregulation of BiP in the pathogenesis of ALS by using mSOD1(G93A) transgenic mice, which were crossed with mice harboring a partial genetic deletion of the BiP gene (BiP^+/−^ mice) (Note that very early embryonic lethality occurs in BiP^−/−^ mice [26]) to generate double mutants (mSOD1/BiP^+/−^ mice), in which we analyzed the progression of the pathological phenotype. We also investigated this issue in a PD model through evaluating the consequences of unilateral lesions carried out in BiP^+/−^ mice with the parkinsonian neurotoxin 6-OHDA, again with the objective to assess neurotoxin susceptibility in these mice compared to wildtype animals. The hypothesis derived from both paradigms is that the neuroprotective effects associated with the activation of the CB_1_ receptor in experimental ALS [18,19,20], as well as those described in experimental PD [15,16,17], could be related to a modulation of CB_1_ receptor function by BiP, which would be conceivable for their recently-demonstrated physical interaction [22], but this will be the objective of future work. In the present study, we wanted to set up a proof of concept that inducing ALS and/or PD pathology in mice having dysregulated BiP function would be followed by alterations in the progression of these two diseases.

## 2. Results

### 2.1. Studies in Experimental ALS: Generation of Double Mutants (mSOD1/BiP^+/−^ Mice)

**Figure 1:**
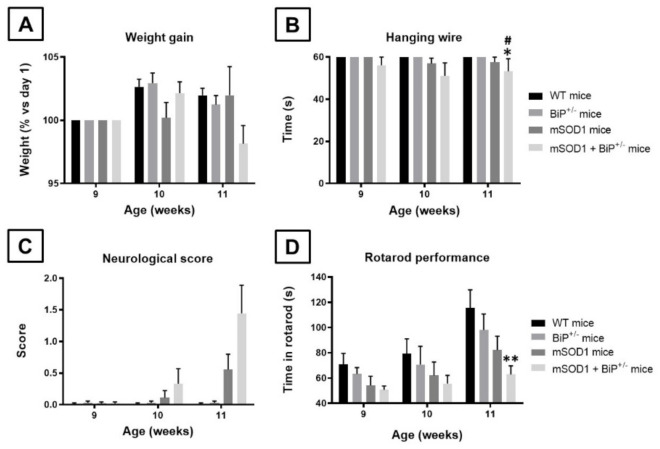
Weight gain (panel (**A**)), hanging wire response (panel (**B**)), neurological score (panel (**C**)), and rotarod performance (panel (**D**)) analyzed at the period of 9 to 11 weeks of age in mSOD1 transgenic and wild-type male mice with normal or partial ablation of the BiP protein. Values are means ± SEM of 6–10 animals per group. Data were assessed by two-way analysis of variance (with repeated measures) followed by the Tukey test (* *p* < 0.05, ** *p* < 0.01 vs. WT mice; ^#^ *p* < 0.05 vs. BiP^+/−^ mice).

The first objective of our study was to cross mice having partial genetic deletion of the BiP gene (BiP^+/−^ mice) with mSOD1 transgenic mice, a classic murine genetic model of ALS, to generate double mutants (mSOD1/BiP^+/−^ mice), in which we evaluated the progression of the pathological phenotype. We first analyzed the weight gain in the four genotypes which proved a trend towards a decrease in double mutants compared to the other three genotypes when animals were 11 week-old, reflected in the 2-way ANOVA (with repeated measures) statistics for the variable age (F(2,70) = 6.57, *p* < 0.01) and in its 2-way interaction with the variable genotype (F(6,70) = 2.93, *p* < 0.05), although the effect did not reach statistical significance with the post hoc analysis (Figure 1A). Similar trends at weeks 10 and 11 after birth were also evident for the animal responses in the hanging wire test (age: F(2,70) = 3.15, *p* = 0.066; see Figure 1B), whereas statistically significant differences were evident for the deterioration in the neurological score which was greater in double mutants at week 11 (as a trend at week 10) compared to wildtype and BiP^+/−^ mice (genotype: F(3,70) = 5.95, *p* < 0.005; age: F(2,70) = 15.48, *p* < 0.0001; 2-way interaction: F(6,70) = 7.49, *p* < 0.0001; see Figure 1C). This was also found for the animal response in the rotarod test with the lowest times in the rod at the three ages analyzed always in the double mutants (genotype: F(3,62) = 2.83, ns; age: F(2,62) = 16.41, *p* < 0.0001; 2-way interaction: F(6,62) = 1.20, ns), but reaching statistical significance as compared with wildtype mice only at week 11 (Figure 1D).

**Figure 2:**
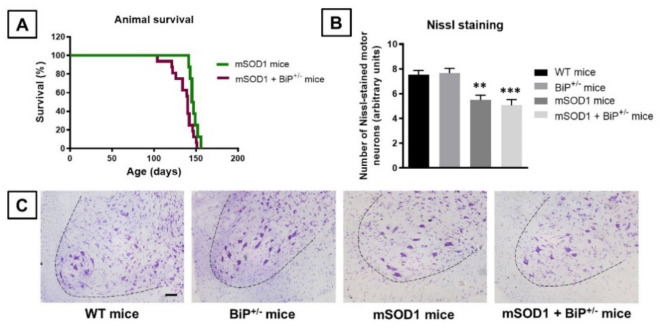
Analysis of animal survival (panel (**A**)) and of the number of Nissl-stained motor neurons (panel (**B**)), including representative images (panel (**C**); scale bar = 100 µm), in the lumbar ventral horn (marked with a dotted line) of the spinal cord in mSOD1 transgenic and wild-type male mice with normal or partial ablation of the BiP protein. In the case of Nissl staining, values are means ± SEM of 5–7 animals per group and were assessed by one-way analysis of variance followed by the Tukey test (** *p* < 0.01, *** *p* < 0.005 vs. WT or BiP^+/−^ mice). Data for animal survival were presented as a Kaplan-Meier plot and assessed by Chi-square test.

This greater neurological worsening seen in the double mutants was also accompanied by a faster mortality compared to mutant SOD1 transgenic mice (χ^2^ = 4.952, *p* < 0.05; Figure 2A). Mortality was initiated in mSOD1/BiP^+/−^ mice at 15 weeks of age with all animals dying at 21 weeks of age (median survival at 140 days), whereas, in mSOD1 mice, mortality was initiated at 20 weeks with all animals dying at 23 weeks (median survival at 146 days) (Figure 2A).

Next, we analyzed several histopathological markers in the ventral horn of the spinal cord, where the cell bodies of lower spinal motor neurons affected in ALS are located. We used Nissl staining to label these neurons, and Iba-1 and GFAP immunofluorescence to identify microglial and astroglial cells, respectively. Our data indicated that the number of Nissl-stained motor neurons was significantly reduced in mSOD1 mice (*p* < 0.01), with a relatively similar reduction in mSOD1/BiP^+/−^ mice (*p* < 0.005), compared with the two control genotypes (F(3,29) = 10.74, *p* < 0.0001; Figure 2B,C).

**Figure 3:**
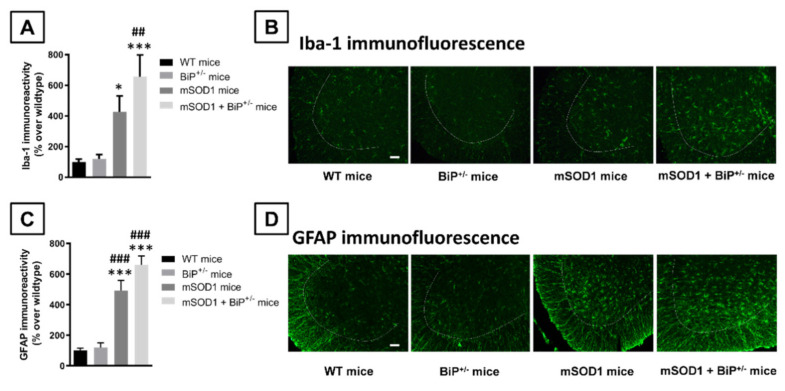
Quantification of Iba-1 (panel (**A**)) and GFAP (panel (**C**)) immunoreactivity, including representative images (panel (**B**) and (**D**), respectively; scale bar = 100 µm), in the lumbar ventral horn (marked with a dotted line) of the spinal cord in mSOD1 transgenic and wild-type male mice with normal or partial ablation of the BiP protein. Values are means ± SEM of 5–7 animals *per* group. Data were assessed by one-way analysis of variance followed by the Tukey test (* *p* < 0.05, *** *p* < 0.005 versus WT mice; ^##^ *p* < 0.01, ^###^ *p* < 0.005 vs. BiP^+/−^ mice).

**Figure 4:**
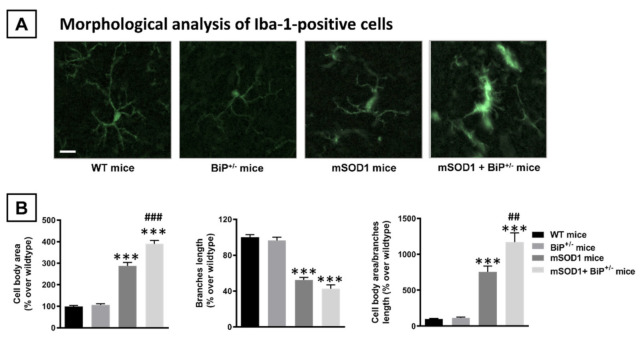
Representative images of Iba-1-positive cells (panel (**A**); scale bar = 25 µm) detected in the spinal cord (lumbar ventral horn) of mSOD1 transgenic and wild-type male mice with normal or partial ablation of the BiP protein, and their morphological analysis (cell body area, length of branches and their ratio; panel (**B**)). Values are means ± SEM of 5–7 animals *per* group. Data were assessed by one-way analysis of variance followed by the Tukey test (*** *p* < 0.005 vs. WT and BiP^+/−^ mice; ^##^ *p* < 0.01, ^###^ *p* < 0.005 vs. mSOD1 mice).

Such important loss of Nissl-stained motor neurons was associated, as expected, with elevated levels of microglial reactivity (Iba-1 immunolabelling) in both mSOD1 genotypes compared to the two control groups (F(3,29) = 8.90, *p* < 0.0005; Figure 3A,B). However, in this case, the immunoreactivity detected in mSOD1/BiP^+/−^ mice showed a numerical trend towards to be higher than in mSOD1 mice having normal expression of BiP (different probability levels compared to control genotypes; Figure 3A,B). The analysis of morphological characteristics of Iba-1-positive cells revealed a higher presence of activated cells, characterized by reduced length in branches (F(3,27) = 77.9, *p* < 0.0001) and elevated cell body area (F(3,27) = 143.3, *p* < 0.0001 and their ratio with branches length (F(3,27) = 52.03, *p* < 0.0001), in mSOD1/BiP^+/−^ mice compared to mSOD1 animals with normal BiP expression, with the activated cells being residual in wildtype genotypes (Figure 4A,B).

**Figure 5:**
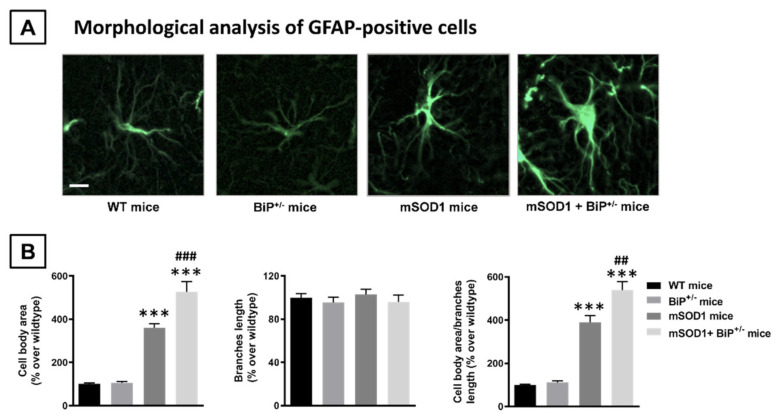
Representative images of GFAP-positive cells (panel (**A**); scale bar = 25 µm) detected in the spinal cord (lumbar ventral horn) of mSOD1 transgenic and wild-type male mice with normal or partial ablation of the BiP protein, and their morphological analysis (cell body area, length of branches and their ratio; panel (**B**)). Values are means ± SEM of 5–7 animals *per* group. Data were assessed by one-way analysis of variance followed by the Tukey test (*** *p* < 0.005 vs. WT and BiP^+/−^ mice; ^##^ *p* < 0.01, ^###^ *p* < 0.005 vs. mSOD1 mice).

Similar results were also found for astroglial reactivity as measured with GFAP immunostaining (F(3,30) = 28.85, *p* < 0.0001; Figure 3C,D), which also tended to be higher in mSOD1/BiP^+/−^ mice compared to the elevation found in mSOD1 mice, although the probability levels (*p* values) with respect to corresponding control genotypes were similar in both cases. Again, the analysis of morphological characteristics of GFAP-positive cells also revealed a higher presence of activated cells, characterized by elevated cell body area (F(3,29) = 65.25, *p* < 0.0001) and their ratio with branches length (F(3,29) = 71.13, *p* < 0.0001), in mSOD1/BiP^+/−^ mice compared to mSOD1 animals with normal BiP expression, with the activated cells being residual in wildtype genotypes (Figure 5A,B).

**Figure 6:**
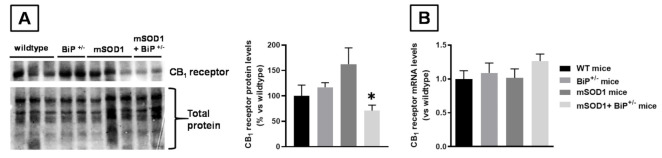
CB_1_ receptor protein (panel (**A**)) and mRNA (panel (**B**)) levels measured in the spinal cord of mSOD1 transgenic and wild-type male mice with normal or partial ablation of the BiP protein. Values are means ± SEM of 5–7 animals per group. Data were assessed by one-way analysis of variance followed by the Tukey test (* *p* < 0.05 vs. mSOD1 mice).

Given the recently demonstrated interaction of BiP protein with the CB_1_ receptor [22], we also wanted to analyze the gene expression and protein levels of this receptor in the spinal cord in the four experimental groups. Whereas we found no differences in mRNA levels (Figure 6B), our data obtained with western blotting demonstrated significantly lower levels found in mSOD1/BiP^+/−^ mice compared to mSOD1 mice (Figure 6A).

### 2.2. Studies in Experimental PD: Unilateral 6-OHDA Lesions in Wildtype and BiP^+/−^ Mice

**Figure 7:**
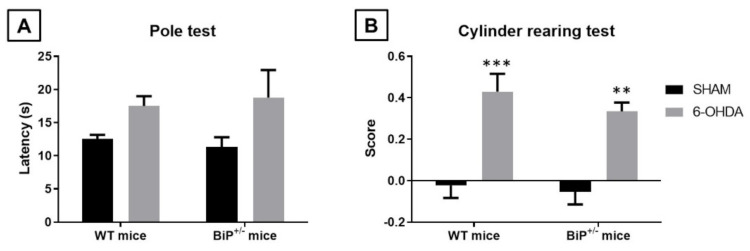
Response in the pole test (panel (**A**)) and in the cylinder rearing test (panel (**B**)) of wild-type male mice with normal or partial ablation of the BiP protein subjected to unilateral 6-OHDA lesions or sham-operated. Values are means ± SEM of more than 5 animals *per* group. Data were assessed by two-way ANOVA followed by the Bonferroni test (** *p* < 0.01, *** *p* < 0.005 vs. the corresponding sham-operated group).

The next objective of our study was to determine whether similar effects to those found upon BiP heterozigosity in experimental ALS translate to other neurodegenerative disorder, such as PD, in which the activation of the CB_1_ receptor is also neuroprotective [15,16,17]. To this end, BiP^+/−^ mice were unilaterally lesioned with the Parkinsonian neurotoxin 6-OHDA with the purpose of confirming a possible greater neurotoxin susceptibility in these mice compared to wildtype animals. Our data, however, did not support that this was the case, as the behavioral and histopathological alterations caused by 6-OHDA were of similar magnitude in BiP^+/−^ mice compared with wildtype animals. Thus, we first analyzed the animal response in the pole test, which resulted to be elevated by the lesion (F(1,30) = 5.994, *p* < 0.05), but showing the same extent for both genotypes (F(1,30) = 0.0003, ns; 2-way interaction: F(1,30) = 0.24, ns; Figure 7A). The same situation was evident for the cylinder rearing test, which proved the expected increase in the hemiparesis shown by lesioned animals (F(1,30) = 34.43, *p* < 0.0001), but again at the same extent in both genotypes (F(1,30) = 0.77, ns; 2-way interaction: F(1,30) = 0.20, ns; Figure 7B).

**Figure 8:**
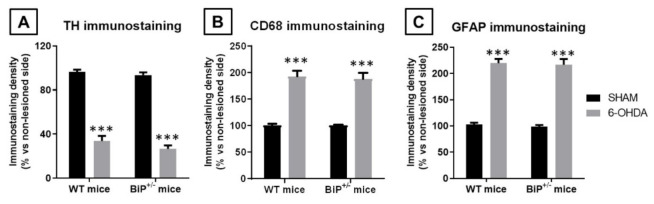
Immunoreactivity for TH (panel (**A**)), CD68 (panel (**B**)) and GFAP (panel (**C**)) measured in a selected area of the substantia nigra *pars compacta* of wild-type male mice with normal or partial ablation of the BiP protein subjected to unilateral 6-OHDA lesions or sham-operated. Values correspond to % of the ipsilateral lesioned side vs. contralateral non-lesioned side and were expressed as means ± SEM of more than 5 animals per group. Data were assessed by two-way ANOVA followed by the Bonferroni test (*** *p* < 0.005 vs. the corresponding sham-operated group).

The lack of differences in the motor defects exhibited by both genotypes after the 6-OHDA lesion correlated with their reduction in tyrosine hydroxylase immunoreactivity in the substantia nigra in the lesioned side (compared to the contralateral non-lesioned side; F(1,30) = 312.9, *p* < 0.0001), which resulted to be similar in both genotypes (F(1,30) = 2.01, ns; 2-way interaction: F(1,30) = 0.35, ns; Figure 8A). Similar results were obtained for the microglial reactivity (measured in this case with CD68 immunostaining) caused by 6-OHDA in the substantia nigra (F(1,30) = 88.17, *p* < 0.0001), which again reacted equally in both genotypes (F(1,30) = 0.07, ns; 2-way interaction: F(1,30) = 0.05, ns; Figure 8B), and also for the elevation of GFAP immunoreactivity, which reflects astrogliosis (F(1,30) = 204.8, *p* < 0.0001), once again with no observable differences across genotypes (F(1,30) = 0.16, ns; 2-way interaction: F(1,30) = 0.002, ns; Figure 8C).

## 3. Discussion

Our general objective in this study was to investigate the consequences of a heterozygous deficiency of the BiP protein, which, by potentially acting as an interacting protein, has been recently associated with CB_1_ receptor function [22], in two neurodegenerative disorders in which the activation of this receptor is known to be neuroprotective: ALS [18,19,20] and PD [15,16,17]. To this end, we used an experimental approach consisting on inducing experimental ALS or PD in mice having a partial deletion in the BiP gene and their corresponding wildtype littermates, and recording the development of the pathological phenotype to determine whether BiP-deficient mice were more vulnerable or not to these experimental insults. Our data showed that the response was different in ALS (greater vulnerability) compared to PD (no differences), which may indicate differences in the role played by BiP in relation with its canonical functions (i.e., control of protein folding and assembly, endoplasmic reticulum stress response) and, eventually, with CB_1_ receptor signaling in those neuronal subpopulations present in the CNS structures affected in both disorders.

In the case of ALS, our experimental approach consisted on generating a double mutant mouse model having the partial deficiency in BiP protein and the G93A mutant form found in the human *SOD1* gene, whose behavioral and histopathological abnormalities were compared first with mSOD1 mice and also with the two control genotypes (wildtype or BiP^+/−^ mice). Our data reveled a clear worsening in the neurological decline, as well as in the behavioral responses in specific motor tests, in particular, the rotarod test, in the double mutants, fueling our hypothesis that dysregulation or deficiency in BiP protein may affect the endogenous protective role exerted by the CB_1_ receptor as activated by the endogenous cannabinoid tone, then promoting a faster neurological decline in the double mutants. Our histopathological data obtained once animals were euthanized at the age showing the maximal neurological deterioration revealed that this was accompanied by higher levels of glial reactivity and acquisition of an activated phenotype, in particular in microglial cells but also in astrocytes, in the ventral horn of the spinal cord at lumbar levels. However, we could not demonstrate that such greater reactive gliosis was associated with also a greater extent in the loss of motor neurons in the spinal cord at the age at which animals were euthanized. It is possible that this response may take place at later phases in parallel to a worsening in the neurological decline, but this will remain to be investigated in further studies. Additionally, pending of future studies is testing that the faster progression found in the pathological phenotype in double mutants is a consequence of the loss of CB_1_ receptor-mediated neuroprotective effects linked in ALS with BiP function as interacting protein. It is important to remark that this protein interacts with many other cellular proteins and that its dysregulation has been already associated with ALS pathogenesis by mechanisms that a priori do not involve the participation of the CB_1_ receptor [23,24,25], so the issue will require additional research that may test a cause–effect relationship between dysregulation in BiP function and loss of CB_1_-mediated neuroprotective effects, and *vice versa*. We can anticipate here that CB_1_ receptor gene expression resulted to be similar in the spinal cord of wildtype or mSOD1 mice with normal or partial ablation of BiP protein, but this result was relatively expected as the recent data indicate that BiP serves as an interacting protein for the CB_1_ receptor [22], so the defects in this protein should affect primarily the signaling for this receptor rather than its gene expression. In fact, the analysis of protein levels for the CB_1_ receptor proved lower levels in the double mutants compared to mSOD1 mice, which may be associated with these defects in receptor signaling, although this will require additional research.

The results were different in the case of PD, the other disorder investigated in this study. In this case, we followed a different experimental strategy, using unilateral 6-OHDA striatal lesions in both BiP^+/−^ and wildtype mice and quantifying the consequences of this lesion at behavioral and histopathological levels. We were unable to detect any differences in the response of animals having a partial deficiency in BiP protein with respect to their controls in two classic parkinsonian motor tests, and the same happened with the analysis of the lesioned substantia nigra in relation with the status of tyrosine hydroxylase-positive neurons and the glial reactivity. Such observation contrasts with previous studies that situated BiP as a neuroprotectant factor in PD acting through mechanisms that involve UPR pathways [27], but these studies were conducted in PD models based on the formation of α-synuclein aggregates, which is not the case with 6-OHDA lesions. This may be an important difference in relation with the ALS model based on mSOD1 (as well as additional models based on the RNA-binding protein TDP-43), which form protein aggregates then facilitating the involvement of BiP [28]. It is also possible that the differences in BiP involvement between PD and ALS may be related to the proposed role of this protein as an interacting protein for CB_1_ receptors, which may be different in those neuronal subpopulations affected in PD with respect to those affected in ALS, a fact that would also require further investigation.

## 4. Materials and Methods

### 4.1. Animals, Experiments and Sampling

Experiments were conducted with two mouse colonies: (i) B6SJL-Tg(SOD1*G93A)1Gur/J transgenic (mSOD1 mice) and non-transgenic littermate sibling mice bred in our animal facilities from initial breeders provided by Dr. Rosario Osta (LagenBio-Ingen, University of Zaragoza, Spain), and (ii) B6.129(Cg)-Hspa5^tm1.1Alee/J^ mice (BiP^+/−^ mice) purchased to Jackson Laboratories (Bar Harbor, ME, USA). Both colonies were housed in a room with controlled photoperiod (08:00–20:00 light) and temperature (22 ± 1 °C) with free access to standard diet and water. All animal experiments were conducted according to local and European rules (directive 2010/63/EU), as well as conformed to ARRIVE guidelines. They were approved by the ethical committees of our university and the regulatory institution (ref. PROEX 059/16).

In a first experiment, mutant SOD1(G93A) transgenic mice were mated with BiP^+/−^ mice (Note that these two mouse colonies were in different backgrounds, so to avoid that this may affect the experiment, only F1 littermates were analyzed) to generate four genotypes to be investigated: (i) wildtype mice with normal expression of the BiP protein (referred to as WT mice); (ii) wildtype mice with heterozygous deletion of the BiP protein (referred to as BiP^+/−^ mice); (iii) mSOD1(G93A) transgenic mice with normal expression of the BiP protein (referred to as mSOD1 mice); and (iv) mSOD1(G93A) transgenic mice with partial genetic ablation of the BiP protein (referred to as mSOD1/BiP^+/−^ mice). All male mice generated were genotyped for the presence or absence of the transgene containing the SOD1^G93A^ mutation (protocol provided by LagenBio-Ingen), and the presence or partial absence of BIP expression (protocol provided by Jackson Laboratories), and were assigned to the four groups according to their genotype. When animals reached 9 weeks of age (at this age mSOD1 mice are still presymptomatic or early symptomatic [29]), they were weekly (9, 10, and 11 weeks) weighted and subjected to three different behavioral analyses (neurological score, performance in the rotarod and the hanging wire tests) designed to record the progression of their pathological phenotype at the neurological level. Afterwards, all animals in the four genotypes were euthanized by rapid decapitation and their spinal cords were rapidly removed. The spinal samples (lumbar area) to be used for histology were fixed for one day at 4 °C in fresh 4% paraformaldehyde prepared in 0.1 M phosphate buffered-saline (PBS), pH 7.4. Samples were cryoprotected by immersion in a 30% sucrose solution for a further day, and finally stored at −80 °C for Nissl staining and immunohistochemical analysis. The spinal samples (also lumbar area) to be used for biochemistry were rapidly frozen by immersion in cold 2-methylbutane, and stored at −80 °C for qPCR or western blot analysis. In a separate study, mice of the two above mSOD1 genotypes (BiP^+/+^ or BiP^+/−^) were used to determine their differences in animal survival, using the following criteria to trigger euthanasia: (i) severe weight loss (>25%); (ii) animals having bristly hair, closed eyes, lethargy or immobility; (iii) paralysis in both hind limbs; and (iv) inability to walk and lack of response to manipulation.

In a second experiment, male C57BL/6 wildtype and BIP^+/−^ mice were used at adult age (3–4-month-old; 25–30 g weight) for stereotaxic unilateral application of 6-OHDA or saline [30]. To do that, mice were anaesthetized (ketamine 40 mg/kg + xylazine 4 mg/kg, i.p.) 30 min after pretreatment with desipramine (25 mg/kg, i.p.), and then 6-OHDA free base (2 μL at a concentration of 2 μg/μL saline in 0.2% ascorbate to avoid oxidation) or saline (for control mice) were injected stereotaxically into the right striatum at a rate of 0.5 μL/min, using the following coordinates: +0.4 mm AP, ±1.8 mm ML and −3.5 mm DV, as described in [31]. Once injected, the needle was left in place for 5 min before being slowly withdrawn, thus avoiding reflux and a rapid increase in intracranial pressure. Control animals were sham-operated and injected with 2 μL of saline using the same coordinates. The lesions were generated using unilateral injection, the advantage of which is that contralateral structures serve as controls for the different analyses. Two weeks after the 6-OHDA lesion or the sham-operation, all animals were analyzed in the pole test and the cylinder rearing test, at the end of which animals were killed by rapid and careful decapitation and their brains were rapidly removed. Brains were fixed for one day at 4 °C in fresh 4% paraformaldehyde prepared in 0.1 M PBS, pH 7.4. Samples were cryoprotected by immersion in a 30% sucrose solution for a further day, and finally stored at −80 °C for immunohistochemical analysis in the substantia nigra.

### 4.2. Behavioral Recording

*Neurological score.* Mice were evaluated for neurological decline using a numerical scale published previously [29] with modifications. The scale ranged from 0 to 15 distributed in three sub-scales (0–5) concentrated on ambulation, strength analysis and hind-foot reflex test. A final score = 0 corresponds to animals which are not symptomatic, whereas a score = 15 reflects a state of total functional loss in hindlimbs and postural control. The assessment of ambulation was carried out placing the animal inside a corridor (10 × 10 × 80 cm), while evaluating postural control and the way in which hindlimbs were leaned during motion. The strength test evaluated the animal ability to drag and offer resistance when the tail was pulled softly to the opposite direction in which the animal moves. Lastly, the hind-foot reflex test evaluated the stiffness of the limbs and their coordination when the mouse was suspended by the tail 10 cm over the surface. The final score was calculated from the sum of values reached in each sub-scale.

Rotarod test. Mice were evaluated for possible motor weakness using the rotarod test, using a LE8200 device (Panlab, Barcelona, Spain). Mice were exposed to a period of acclimation and training (first session: 0 r.p.m. for 30 s; second and third sessions: 4 r.p.m. for 60 s, with periods of 10 min between sessions), followed 30 min later by the assay. Mice were placed into the apparatus and the rotational speed was increased from 4 to 40 r.p.m. over a period of 300 s to measure the time to fall off. Mice were tested for 3 consecutive trials with a rest period of approximately 15 min between trials and the mean of the 3 trials was calculated.

Hanging wire test. The latency of mice to fall from a wire cage top, which was slowly inverted and suspended at approximately 30 cm to the floor, was also used as an index of motor weakness. The test was repeated three times to obtain the mean value of the three trials.

Pole test. Mice were placed head-upward on the top of a vertical rough-surfaced pole (diameter 8 mm; height 55 cm) and the time until animals descended to the floor was recorded with a maximum duration of 120 s. When the mouse was not able to turn downward and instead dropped from the pole, the time was taken as 120 s (default value).

Cylinder rearing test. Given that the lesion was unilateral in the experiment with 6-OHDA, this test attempted to quantify the degree of forepaw (ipsilateral, contralateral, or both) preference for wall contacts after placing the mouse in a methacrylate transparent cylinder (diameter: 15.5 cm; height: 12.7 cm; [32]. Each score was made out of a 3 min trial with a minimum of 4 wall contacts.

### 4.3. Histological Procedures

Tissue slicing. In the ALS experiment, fixed spinal cords were sliced with a cryostat at the lumbar level (L4–L6) to obtain coronal sections (20 μm thick) that were collected on gelatin-coated slides. Sections were used for procedures of Nissl-staining and immunofluorescence. In the PD experiment, brains were sliced in coronal sections (containing the substantia nigra) in a cryostat (30 µm thick) and collected on antifreeze solution (glycerol/ethylene glycol/PBS; 2:3:5) and stored at −20 °C until used. Sections were mounted on gelatin-coated slides.

Nissl staining. Slices were used for Nissl staining using cresyl violet, as previously described [33], which permitted to determine the effects of particular treatments on cell number. A Leica DMRB microscope (Leica, Wetzlar, Germany) and a DFC300Fx camera (Leica) were used to study and photograph the tissue, respectively. To count the number of Nissl-stained motor neurons (>400 μm^2^) in the ventral horn, high-resolution photomicrographs were taken with a 10× objective under the same conditions of light, brightness, and contrast. Counting was carried out with ImageJ software (U.S. National Institutes of Health, Bethesda, MD, USA, http://imagej.nih.gov/ij/, 1997–2012). At least 6 images *per* animal were analyzed to establish the mean of all animals studied in each group. Analyses were always conducted by experimenters who were blinded to all animal characteristics. Data were expressed as arbitrary units.

Immunofluorescence analysis in the ALS experiment. Spinal slices were used for detection and quantification of GFAP or Iba-1 immunofluorescence. After preincubation for 1 h with Tris-buffered saline with 0.1% Triton X-100 (pH 7.5), sections were sequentially incubated overnight at 4 °C with the following polyclonal antibodies: (i) anti-Iba-1 (Wako Chemicals, Richmond, VI, USA) used at 1:500; or (ii) anti-GFAP (Dako Cytomation, Glostrup, Denmark) used at 1:200, followed by washing in Tris-buffered saline and a new incubation (at 37 °C for 2 h) with an anti-rabbit secondary antibody conjugated with Alexa 488 (Invitrogen, Carlsbad, CA, USA). A DMRB microscope and a DFC300Fx camera (Leica, Wetzlar, Germany) were used for slide observation and photography. The mean density of immunolabelling was measured in the selected areas using at least 6 sections *per* animal. In all analyses, data were transformed to the percentage over the mean obtained in the wild-type group for each parameter. For quantification of the glial activation state, we classified GFAP- or Iba-1-positive cells in resting or activated based on morphological criteria published previously [34], which consist in calculating the ratio between cell body area and cell process length.

Immunostaining analysis in the PD experiment. Brain sections containing the substantia nigra were mounted on gelatin-coated slides, and, once adhered, washed in 0.1 M potassium PBS (KPBS) at pH 7.4. Endogenous peroxidase was blocked by 30 min incubation at room temperature in peroxidase blocking solution (Dako Cytomation, Glostrup, Denmark). After several washes with KPBS, sections were incubated overnight at room temperature with the following polyclonal antibodies: (i) rabbit anti-TH (Chemicon-Millipore, Temecula, CA, USA) used at 1/200; (ii) rat anti-mouse CD68 antibody (AbD Serotec, Oxford, UK) used at 1/200; or (iii) rabbit anti-mouse GFAP antibody (Dako Cytomation, Glostrup, Denmark) used at 1/200. Dilutions were carried out in KPBS containing 2% bovine serum albumin and 0.1% Triton X-100 (Sigma Chem., Madrid, Spain). After incubation, sections were washed in KPBS, followed by incubation with the corresponding biotinylated secondary antibody (1/200) (Vector Laboratories, Burlingame, CA, USA) for 1 h at room temperature. An avidin–biotin complex (Vector Laboratories, Burlingame, CA, USA) and 3,3′-diaminobenzidine substrate–chromogen system (Dako Cytomation, Glostrup, Denmark) were used to obtain a visible reaction product. Negative control sections were obtained using the same protocol with omission of the primary antibody. A Leica DMRB microscope and a DFC300FX camera (Leica, Wetzlar, Germany) were used for the observation and photography of the slides, respectively. For quantification of TH, GFAP or CD68 immunostaining in the substantia nigra, we used the NIH Image Processing and Analysis software (ImageJ; NIH, Bethesda, MD, USA) using 4–5 sections, separated approximately by 200 µm, and observed with 5×–20× objectives depending on the method and the brain area under quantification. In all sections (a minimum of 6 *per* animal were used), the same area of the substantia nigra *pars compacta* were analyzed. Analyses were always conducted by experimenters who were blinded to all animal characteristics. Data were expressed as percentage of immunostaining intensity in the ipsilateral (lesioned) side over the contralateral (non-lesioned) side.

### 4.4. Real Time RT-qPCR Analysis

Total RNA was extracted from tissues using Trizol (Life Technologies, Alcobendas, Spain). The total amount of RNA extracted was quantified by spectrometry at 260 nm and its purity was calculated as the ratio between the absorbance values at 260 and 280 nm. RNA integrity was confirmed in agarose gels. DNA was removed and single-stranded complementary DNA was synthesized from 0.8 μg of total RNA using a commercial kit (Rneasy Mini Quantitect Reverse Transcription, Qiagen, Izasa, Madrid, Spain). The reaction mixture was kept frozen at −20 °C until enzymatic amplification. Quantitative real-time PCR assays were performed using TaqMan Gene Expression Assays (Applied Biosystems, Foster City, CA, USA) to quantify mRNA levels for CB_1_ receptor (Mm00432621_s1), using GAPDH expression (Mm99999915_g1) as an endogenous control gene for normalization. The PCR assay was performed using the 7300 Fast Real-Time PCR System (Applied Biosystems, Foster City, CA, USA) and the threshold cycle (Ct) was calculated by the instrument’s software (7300 Fast System, Applied Biosystems, Foster City, CA, USA). Expression levels were calculated using the 2^−ΔΔCt^ method, but, for presentation, data were transformed to the percentage over the mean obtained in the wild-type group for each parameter.

### 4.5. Western Blot Analysis

Frozen tissues were homogenized in an ice-cold radioimmunoprecipitation assay (RIPA) buffer for protein extraction. Homogenates were centrifuged at 10,000× *g* for 15 min at 4 °C. Bio-Rad DC protein assay kit (Bio-Rad Laboratories, CA, USA) was used to quantify protein concentration, using bovine serum albumin (BSA) as the standard protein. Then, 15 μg of protein were boiled for 5 min in Laemmli SDS loading buffer (10% glycerol, 5% SDS, 5% β-mercaptoethanol, 0.01% bromophenol blue, and 125 mM TRIS-HCl, pH 6.8) and loaded onto a 12% acrylamide gel (TGX Stain-free Gel FastCast; Bio-Rad Laboratories, CA, USA). After electrophoresis, proteins were transferred to PVDF membranes (Immobilon-P, Millipore, MA, USA) using mini Trans-Blot Electrophoretic transfer cell (Bio-Rad Laboratories, CA, USA). Membranes were then blocked for 1 h at room temperature with Tris-buffered saline containing 5% BSA and 0.1% Tween-20, and incubated overnight at 4 °C with the anti-CB1 polyclonal antibody (CB1-Rb-Af380, Frontier Institute, Hokkaido, Japan) used at 1/10,000. Membranes were finally incubated with an ECL horseradish peroxidase-linked whole secondary antibody (GE Healthcare UK Limited, Buckinghamshire, UK) used at a 1/10,000 dilution for 1 h at room temperature. Reactive bands were detected by chemiluminescence with the Amersham ECL Prime Western Blotting Detection Reagent (GE Healthcare UK Limited, Buckinghamshire, UK). Images were analyzed with Image Lab software (Bio-Rad Laboratories, CA, USA). Data were calculated as the ratio between the optical densities of the specific protein band and the total protein measured in membranes, and then normalized as percentages over the values of wild-type mice.

### 4.6. Statistics

Data were assessed using one-way or two-way ANOVA (with repeated measures, if required) followed by the Tukey or the Bonferroni test, as required, using GraphPad Prism, version 8.00 for Windows (GraphPad Software, San Diego, CA, USA). Survival data were assessed using Log-Rank test and presented with a Kaplan-Meier analysis. A p value lower than 0.05 was used as the limit for statistical significance. The sample sizes in the different experimental groups were always ≥5.

## 5. Conclusions

Therefore, our data suggest that a dysregulation in BiP protein may possibly contribute to ALS pathogenesis, but this did not occur in experimental PD, or at least not in the model of PD used in this study. The hypothesis derived from both paradigms is that the neuroprotective effects associated with the activation of the CB_1_ receptor in experimental ALS [18,19,20], but not those found in experimental PD [15,16,17], might be modulated by BiP function, a notion that will require additional research. Preliminary data obtained here may anticipate that this is the case in ALS, as CB_1_ receptor levels were found to be significantly reduced in mSOD1 mice having partial deletion of BiP gene.

## Data Availability

Data supporting reported results may be supplied upon request by authors.
